# Supplementary figures and images for: A survey on exponential random graph models: an application perspective
Source: PeerJ Comput Sci. 2020 Apr 6;6:e269. doi: 10.7717/peerj-cs.269 (PMC7924687; doi:10.7717/peerj-cs.269)

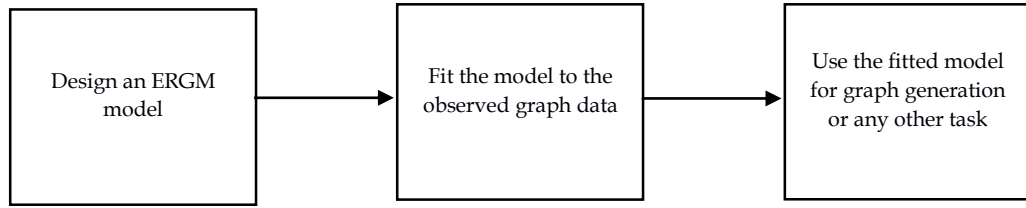

Supplement: Figure S1 [file peerj-cs-06-269-s001.pdf]

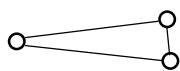

1-triangle

(a)

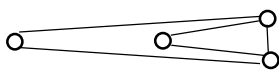

2-triangle

(b)

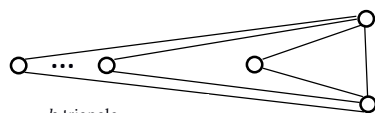

$k$ -triangle

(c)

Supplement: Figure S2 [file peerj-cs-06-269-s002.pdf]

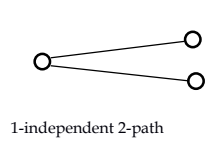

(a)

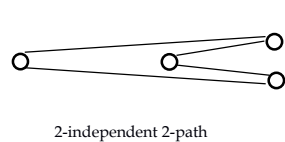

(b)

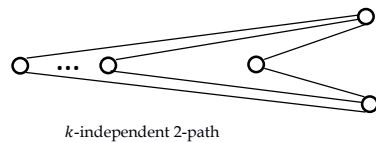

(c)

Supplement: Figure S3 [file peerj-cs-06-269-s003.pdf]
