# Supplementary material for: A survey on exponential random graph models: an application perspective
Source: PeerJ Comput Sci. 2020 Apr 6;6:e269. doi: 10.7717/peerj-cs.269 (PMC7924687; doi:10.7717/peerj-cs.269)
Supplement: Table S1 [file peerj-cs-06-269-s004.docx]

| **Name** | **Figure**  **(if applicable)** | **Specification** |
| --- | --- | --- |
| Edges |  | It shows the number of edges in the graph and controls the graph size. |
| Shared Nodes  Or two path |  | The number of the nodes’ pairs with the same neighbors. |
| Triangle |  | The number of the nodes’ sets of three nodes that form a triangle. |
| Cycles |  | The number of the three or more nodes that form a cycle. |
| Triangle Percentage |  | The number of the triangles in the network divided by the whole number of possible triangles. |
| Density |  | The edge counts divided by the number of all possible edges in an undirected graph. |
