# Supplementary material for: A survey on exponential random graph models: an application perspective
Source: PeerJ Comput Sci. 2020 Apr 6;6:e269. doi: 10.7717/peerj-cs.269 (PMC7924687; doi:10.7717/peerj-cs.269)
Supplement: Table S2 [file peerj-cs-06-269-s005.docx]

| **Name** | **Figure**  **(if applicable)** | **Specification** |
| --- | --- | --- |
| Mutual |  | The number of the nodes’ pairs which both possible edges between them exist. |
| Activity |  | The number of the nodes’ triplets that there is two edge from one of them to the two others. |
| Popularity |  | The number of the nodes’ triplets which two of them are connected to a single one. |
| Transitivity |  | The number of the nodes’ triplets that form a transitivity relation. |
| Triangle |  | The number of three nodes that form a triangle. |
| Cycles |  | The number of the three or more nodes that form a cycle. |
| Asymmetric |  | The number of the two nodes with only one connection from one of them to another. |
| Density |  | The number of the edges divided by the number of all possible edges in a directed graph. |
| Triangle percentage |  | The number of the triangles divided by the number of all possible triangles in a directed graph. |
