# Supplementary material for: A survey on exponential random graph models: an application perspective
Source: PeerJ Comput Sci. 2020 Apr 6;6:e269. doi: 10.7717/peerj-cs.269 (PMC7924687; doi:10.7717/peerj-cs.269)
Supplement: Table S3 [file peerj-cs-06-269-s006.docx]

| **Name** | **Specification** |
| --- | --- |
| Covariate effect | Considering the effect of some nodes attributes as one of the statistics of the network. Sum of both nodes of each present edge is considered as the value of that statistic. |
| Factor attribute effect | The number of repetition of a particular value of some attributes in the network. |
| Homophily | The number of edges with equal value for some attributes. |
| Absolute difference | The absolute difference of both nodes of each present edge is considered as the value of that statistic. |
| Degree | Using the nodes’ degree as a statistic. There are different approaches to incorporate it into a single attribute. |
| $k$-star | The number of configurations that a central node is connected to $k$ number of other distinct nodes. |
| Isolates | The number of nodes with degree zero. |
| Mean vertex degree | Using the average degree of all nodes as a statistic. |
