# Supplementary material for: A survey on exponential random graph models: an application perspective
Source: PeerJ Comput Sci. 2020 Apr 6;6:e269. doi: 10.7717/peerj-cs.269 (PMC7924687; doi:10.7717/peerj-cs.269)
Supplement: Table S4 [file peerj-cs-06-269-s007.docx]

| **Name** | **Specification** |
| --- | --- |
| Covariate effect | Considering the effect of some nodes attributes as one of the statistics of the network. For directed networks, inward node minus outward node attribute of present edges can be used for its value. However, different variations exist. |
| Factor attribute effect | The number of repetition of a particular value of some attributes in the network. |
| Homophily | The number of edges with equal value for some attributes. |
| Difference | The difference between both nodes of each present edge is considered as the value of that statistic. |
| In-degree | Using nodes’ in-degree as a statistic. There are different approaches to incorporate it into a single attribute. |
| Out-degree | Using nodes’ out-degree as a statistic. There are different approaches to incorporate it into a single attribute. |
| Isolate | The number of nodes with degree zero. |
| Mean vertex degree | Using the average degree of all nodes as a statistic. (in-degree, out-degree or both) |
